# Uterine Perforation With a Uterine Balloon Tamponade During the Management of Secondary Postpartum Haemorrhage

**DOI:** 10.7759/cureus.98543

**Published:** 2025-12-05

**Authors:** Mona M Hersi Farah, Meena Nayagam, Magdalena Szybka, Hannah Missfelder-Lobos, Helen Bolton, Norman Shreeve

**Affiliations:** 1 Obstetrics and Gynaecology, Cambridge University Hospitals NHS Foundation Trust, Cambridge, GBR; 2 Family Medicine, North West Anglia NHS Foundation Trust, Cambridge, GBR; 3 Medicine, Cambridge University Hospitals NHS Foundation Trust, Cambridge, GBR; 4 Gynaecologic-Oncology, Cambridge University Hospitals NHS Foundation Trust, Cambridge, GBR

**Keywords:** bakri balloon, ct imaging perforated uterus, midline emergency laparotomy, postpartum haemorrhage (pph), secondary postpartum hemorrhage (pph), uterine perforation

## Abstract

Postpartum haemorrhage (PPH) continues to be a significant global challenge in maternal health. Effective and timely management is essential to prevent severe morbidity and mortality. Postpartum haemorrhage is classified as primary or secondary. Management options include medical, mechanical, and surgical interventions; uterine balloon tamponade is a mechanical method used to control bleeding.

We present an atypical case of secondary PPH in a 36-year-old multiparous woman with an uncomplicated antenatal course who experienced a spontaneous vaginal delivery complicated by manual removal of the placenta and primary PPH with an estimated blood loss of 3500 ml. She later presented with recurrent secondary PPH, prompting transfer to a regional tertiary care centre for further investigation. Imaging and multidisciplinary evaluation revealed uterine perforation associated with a uterine balloon tamponade. Surgical intervention via midline laparotomy was performed for uterine repair.

This case highlights the importance of maintaining a high index of suspicion for uterine injury in persistent or atypical secondary PPH, particularly when mechanical interventions such as balloon tamponade are employed. Early recognition and multidisciplinary management are critical to optimising outcomes. A literature review is included to contextualise this rare but serious complication.

## Introduction

Postpartum haemorrhage (PPH) is defined as a blood loss greater than 500 ml after vaginal birth and greater than 1000 ml after a caesarean section [1]. It can be further classified into primary PPH (within 24 hours of birth) and secondary PPH (from 24 hours until 12 weeks after birth) [1]. Postpartum haemorrhage is the leading cause of maternal mortality globally [2]. The WHO states that of all reported maternal deaths, PPH is responsible for over 20% [2]. In the UK, the 2019-2021 Perinatal Mortality Surveillance Reports (MBRRACE) identified 17 direct deaths due to obstetric haemorrhage [3]. Significant PPH is often preventable and manageable, yet millions of women continue to encounter it each year. While the WHO has provided guidance to address this significant issue, the global maternal mortality ratio remains below its target. Consequently, progress has stagnated in recent times, and the improvement in management of such cases remains a global priority [1,2]. In general, a targeted management protocol should be initiated upon the recognition of PPH, involving pharmacological, mechanical, and surgical interventions, where the accurate identification of the underlying cause is crucial to effectively controlling obstetric haemorrhage.

The most common cause of PPH is uterine atony, accounting for around 80% of all cases [1,4]. In recent times, the intrauterine balloon tamponade, e.g., the Bakri balloon, has emerged as an adjunctive therapy when pharmacological interventions alone are inadequate. Data suggests that the Bakri balloon, as used in the case described in this report, is highly effective in controlling bleeding where uterine atony is suspected. It is believed to be less invasive than other surgical methods, such as brace suture, cost-effective, easy to use, associated with minimal complications, and offers a clear indication of ongoing bleeding through its drainage channel [1,4-6].

However, this case report highlights the importance of considering other less common causes of persistent secondary PPH, particularly where there is an extended postpartum interval. An atypical presentation should raise the clinical suspicion of uterine injury or possible other vascular origin, particularly when there are additional risk factors or unusual circumstances in which a balloon tamponade is used.

## Case presentation

The patient is a 36-year-old female with no significant medical or surgical history, who experienced an unremarkable first pregnancy. In her second pregnancy, too, she had an uncomplicated antenatal course. She then underwent a spontaneous vaginal delivery, which was complicated by the manual removal of the placenta (MROP) and an associated massive primary PPH, where the estimated blood loss was 3500 ml. This was managed with the insertion of a Bakri balloon tamponade (BBT) at her local maternity hospital. Due to significant blood loss, she was subsequently admitted to the ICU for stabilisation and monitoring. The Bakri balloon was removed, and she recovered well. She was discharged home without additional intervention for routine postnatal care in the community.

At 11 days postpartum, the patient presented to her local hospital experiencing significant vaginal bleeding, including the passage of large blood clots. The bleeding settled without further intervention, and the estimated blood loss was 300 ml. Considering this was her first presentation of secondary PPH, conservative management was deemed appropriate, and she was treated with antibiotics and discharged without further imaging.

Two weeks later, the patient suffered another significant episode of vaginal bleeding at home. She was readmitted to her local hospital and immediately transferred to the operating theatre, as the estimated blood loss was around 2500 ml. In the theatre, an examination under anaesthesia (EUA) was performed to identify the source of bleeding and achieve haemostasis. During the EUA, placental tissue was identified within the uterine cavity; however, it was deemed not possible to be removed. A decision was made to insert a BBT, and vaginal packing was also applied to help support the BBT and keep it in situ. During this procedure, the patient received five units of red blood cells and one unit of fresh frozen plasma. Once haemostasis was achieved, she was transferred to the regional tertiary hospital with the expertise and treatment available for abnormal invasive placentas (AIP) and additional treatment options such as interventional radiology, if deemed necessary.

**Figure 1:**
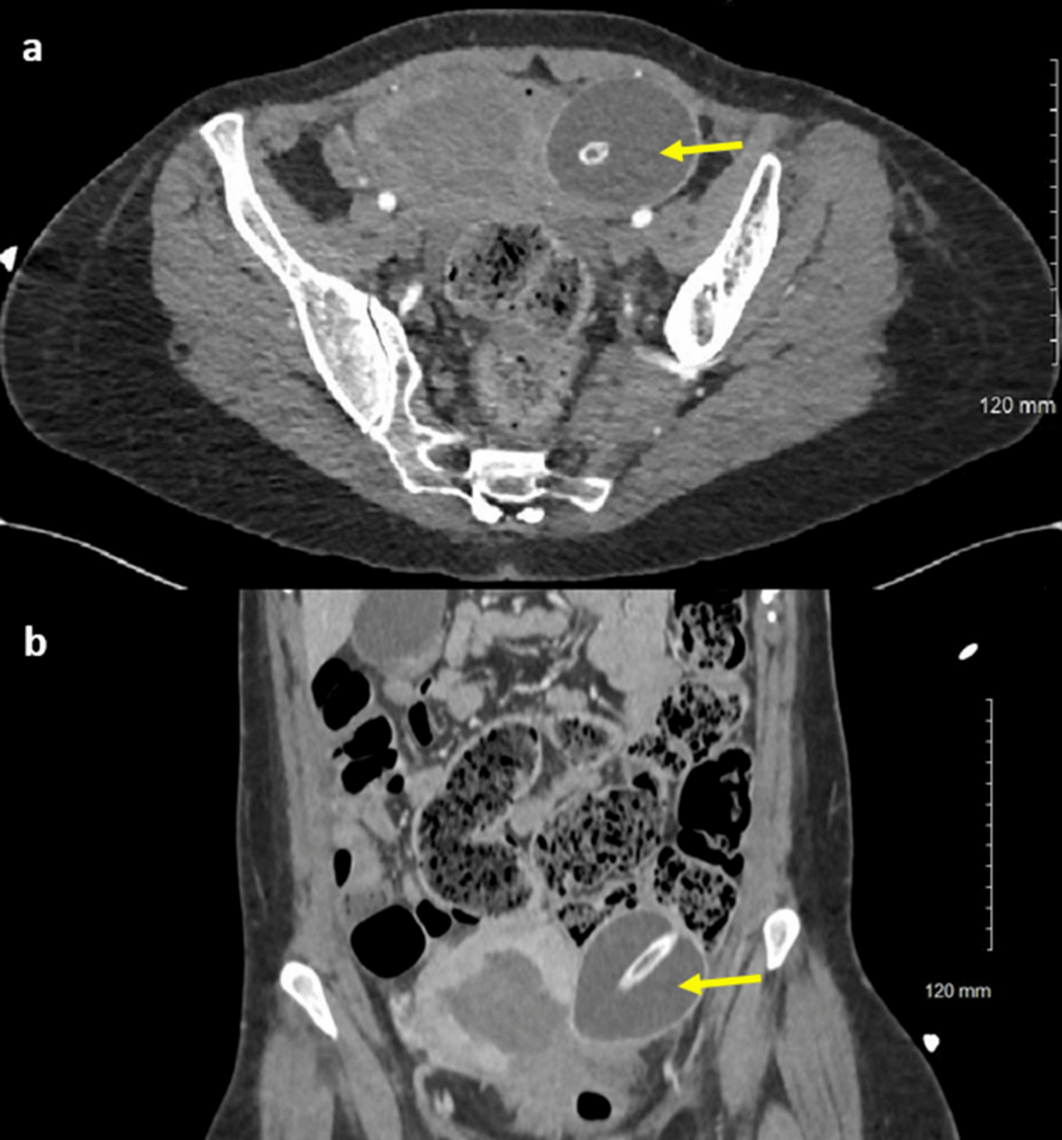
Axial (a) and coronal (b) CT with contrast images taken before transfer to theatre The images show a large defect in the uterine wall with appearances suggestive of a perforated uterine body into the pelvis (yellow arrow).

On arrival at the tertiary unit, the patient was assessed by the on-call obstetric team. She was found to be tender on abdominal palpation, and the suspected diagnosis was initially that the secondary PPH was caused by an AIP, as stated in the referral. The differential diagnosis by the obstetric team also included underlying endometrial infection, retained products of conception, and arteriovenous malformation. Initial haematological investigations indicated a haemoglobin of 108 g/dL, a platelet count of 130 10*9/l, prothrombin time of 13.1 seconds, an activated partial thromboplastin time of 26.3 seconds, and a fibrinogen level of 1.63 g/L. However, in this unusual situation of recurrent massive secondary PPH, further imaging of the pelvis and abdomen by triphasic CT scan was considered so as to define the pelvic anatomy. The imaging showed a perforation of the left lower uterine body, with the BBT device seen passing through the myometrium. A pelvic haematoma was identified, but no active haemorrhage was noted (Figure 1).

**Figure 2:**
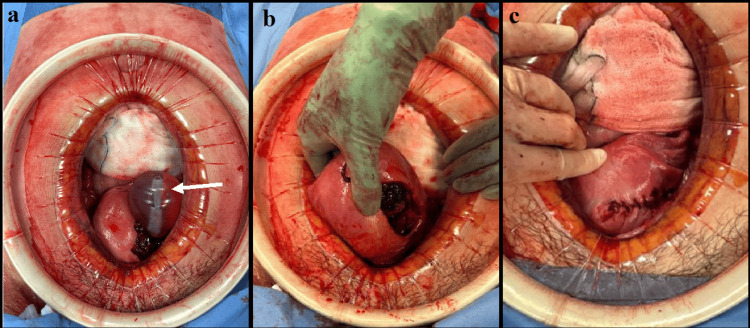
Intraoperative photography of exploratory laparotomy a: Uterine rupture and extra-uterine placement of Bakri balloon (arrow) through the anterior myometrium; b: Removal of the device revealed a large 5 cm myometrial defect with associated necrotic myometrial tissue at the upper medial edge of the perforation, alongside retained products of conception; c: Surgical closure and haemostasis achieved

Following multidisciplinary discussion between the on-call obstetrician, gynaecologist, and interventional radiologist, it was agreed that a midline laparotomy would allow the best possible access for either a uterine repair or hysterectomy when deemed appropriate. As the patient had not completed her family, it was planned that pelvic vessel embolisation with interventional radiology would be attempted ahead of hysterectomy if ongoing bleeding was identified. A midline laparotomy with routine entry was performed, where blood clots were removed from the pelvis. A large uterine perforation with the Bakri balloon perforating through the left anterior wall of the uterus through a defect approximately 5 cm in length was confirmed. The Bakri balloon was deflated and removed vaginally (Figure 2).

Presumed retained placental tissue was removed from the uterine cavity during the procedure and sent for histological examination. The uterine defect was closed with 0-Vicryl sutures to the rupture, taking care to secure both angles in a two-layer repair. No further bleeding was observed, and a pelvic lavage was performed before abdominal closure. The femoral catheter was removed prior to extubation. The histology report demonstrated no definitive placenta tissue, and there was also no evidence of malignancy. Overall, the procedure was successful, and the patient recovered well under antibiotic cover. Approximately nine weeks from transfer to the tertiary centre, a postnatal debrief was held, reviewing the events from transfer to discharge. Discussion included considerations for future pregnancies, with a focus on mode and timing of delivery.

## Discussion

In this case, the complex nature of managing secondary PPH was evident. The multiple risk factors for PPH, including a vaginal delivery complicated by MROP, treatment with antibiotics for a suspected underlying endometrial infection, and retained products of conception, along with both primary and secondary massive bleeding, necessitated the insertion of a Bakri balloon in each instance. We review various aspects that support the use of BBT in such cases.

Use of the Bakri balloon

The Bakri balloon device utilises the 'tamponade test' method. This approach involves inflating the balloon and subsequently assessing the blood loss. If blood loss is controlled after balloon inflation, the test is considered positive; if uncontrolled, it is considered negative, indicating the potential need for further surgical intervention such as laparotomy [2,7,8]. Per instructions, it is recommended to use ultrasound to confirm the placement of the balloon once it has been inflated to the specified volume [9].

A significant advancement in the management of PPH was introduced with the Bakri balloon, the first effective intrauterine device specifically designed for uterine tamponade [7,10,11]. In a pivotal case series published by Bakri et al., six patients were evaluated, including five obstetric patients who presented with persistent haemorrhage. Haemostasis was successfully achieved in all cases [7]. Subsequently, a case series on the use of the Bakri balloon device led to its adoption in maternity units to manage PPH. Additional studies corroborated these findings, revealing that a few years after its implementation, there was a reduction in the rate of laparotomy required to control PPH [7,10-12]. The Scottish Confidential Audit of Severe Maternal Morbidity identified in its report the significant decline of maternal obstetric haemorrhage (MOH) post peripartum hysterectomy over 10 years, and this decline was associated with increasing conservative measures, and in particular, intrauterine balloons [1,13].

Uterine balloon tamponade complications

Leparco et al. described an unrecognised complication associated with the Bakri balloon when inserted during secondary PPH, under both direct and ultrasound guidance. In this case, massive maternal haemorrhage persisted, necessitating a laparotomy that ultimately revealed the inflated Bakri balloon was located within the broad ligament [14]. Similarly, Spencer et al. reported on the perforation of a Bakri balloon into the broad ligament, and Rocher et al. described a case of massive haemoperitoneum due to migration of the Bakri balloon through the broad ligament alongside anterior uterine rupture [15,16].

Whilst relatively uncommon, several additional complications have been reported regarding uterine balloon device usage, such as infection, endometritis, cervical tears, laceration of the lower segment of the vagina, uterine perforation during insertion, or overdistension [17,18]. Bahuguna et al. also reported a case where a Bakri balloon was inserted under ultrasound guidance during a secondary PPH; the patient had complained of pain under the rib and lower abdomen and subsequently developed hypotension. On assessment, it was confirmed that there was a uterine breach. Emergency laparotomy revealed uterine perforation, which was successfully repaired [19].

A review of the literature identified one case that demonstrated successful use of the Bakri balloon in the management of secondary PPH. There is limited evidence to support the use of uterine tamponade in secondary PPH compared with its more established role in primary PPH [7,17,20]. However, the use of ultrasound to guide the insertion of BBT may help reduce complications. For example, in the case reported here, the uterine perforation may have been recognised if the insertion had been performed under direct ultrasound guidance. Germano et al. emphasised the role of ultrasound guidance during the insertion of the Bakri balloon. In their retrospective cohort study, the ultrasound-guided group demonstrated a 99% success rate, highlighting a decrease in the need for Bakri balloon adjustments and more rapid control of PPH [21].

## Conclusions

When taking into account the nature of BBT complications published in the literature and the atypical presentation in this case, the working theory was that the uterine perforation occurred during the first insertion of the Bakri balloon, as supported by the patient's ongoing episodes of recurrent vaginal bleeding that occurred thereafter. However, it is impossible to confirm the exact timing of the occurrence of the perforation. In summary, further investigation and imaging should be prioritised when a patient presents with secondary PPH, especially atypical presentations and in cases of significant and recurrent bleeding. Whilst the role of BBT in primary PPH and cases of suspected uterine atony is well evidenced, we believe that the use of BBT in secondary PPH should be applied with caution. If haemorrhage is ongoing and time is limited, uterine tamponade may be attempted, with Bakri balloon insertion ideally performed under ultrasound guidance by experienced obstetricians. In more stable patients, cross-sectional imaging should precede intervention, and any insertion of the Bakri balloon should be undertaken with heightened caution.
